# Wild-type Measles Virus in Brain Tissue of Children with Subacute Sclerosing Panencephalitis, Argentina

**DOI:** 10.3201/eid0910.030180

**Published:** 2003-10

**Authors:** Paola Roxana Barrero, Jorge Grippo, Mariana Viegas, Alicia Susana Mistchenko

**Affiliations:** * Hospital de Niños Dr. Ricardo Gutiérrez, Buenos Aires, Argentina

## Abstract

We studied eight children who had measles at 6 to 10 months of age during the 1998 Argentine measles outbreak and in whom subacute sclerosing panencephalitis developed 4 years later. We report the genetic characterization of brain tissue–associated measles virus samples from three patients. Phylogenetic relationships clustered these viruses with the wild-type D6 genotype isolated during the 1998 outbreak. The children received measles vaccine; however, vaccinal strains were not found.

Measles is often incorrectly regarded as a mild disease, and priority is not given to measles elimination programs in some countries (*1*). Nevertheless, substantial progress has been made in eliminating measles virus from the Americas through massive vaccination campaigns and maintaining high measles population immunity over time. From 1990 to 1996, measles cases declined from 250,000 to 2,109; however, in 1997, measles reemerged in the Americas, with 70,983 confirmed cases. From the last Argentine outbreak (July 1997–May 1999), 10,354 confirmed measles cases were reported, most of them in unvaccinated preschool-aged children in the greater Buenos Aires metropolitan area (*2*).

Although measles virus (*Morbillivirus* genus, *Paramyxovirus* family) is not highly neurotropic, it can establish long-term persistent infection in brain cells. Three different neurologic complications result from interactions of measles virus with neural tissue. Acute postinfectious encephalitis, usually appearing 5–14 days after the rash, is thought to be a virus-induced autoimmune disease. Measles inclusion–body encephalitis, which occurs in immunocompromised patients after a latent period of 3–6 months, is believed to be a direct measles virus infection of neural tissue. The third form, subacute sclerosing panencephalitis (SSPE), manifests 2–10 years after primary measles infection as a progressive and fatal chronic neurodegenerative disease caused by persistent defective measles virus in neurons and oligodendrocytes. SSPE develops with high titers of anti-measles antibodies both in cerebrospinal fluid (CSF) and serum, which seem unable to eliminate measles virus from the brain (*3*).

Although measles virus is serologically monotypic, genetic variability has defined eight clades, including 20 genotypes and a putative new genotype that are geographically and temporally restricted (*4*). Evidence does not indicate that wild-type measles virus strains differ in terms of either pathogenesis or neurovirulence. Measles virus recovered from patients with SSPE differs from wild-type viruses in a number of mutations that mainly affect the matrix, hemagglutinin (H), nucleocapsid (N), and fusion genes. The matrix accumulates the highest level of mutations in the entire open reading frame; by contrast, the N is modified in the carboxyl terminus, and the H has biased hypermutation in a limited region (*5*,*6*). Despite the paucity of studies in molecular epidemiology of SSPE, measles virus sequences obtained from brain tissues are homologous to the genotype circulating at the time of primary exposure to measles virus (*7*).

We studied eight children who had measles as infants during the 1998 measles outbreak in Argentina and in whom SSPE developed 4 years later. We report the genetic characterization of brain tissue–associated measles virus from three of these patients.

## The Study

Diagnosis of SSPE was based on Dyken’s criteria, which include progressive cognitive decline and stereotypical myoclonus, characteristic electroencephalogram (EEG) changes, raised CSF globulin levels without pleocytosis, raised CSF measles antibody titers, and typical histopathologic findings in brain biopsy materials (*8*).

Antimeasles immunoglobulin (Ig) G antibodies in CSF and serum samples were assessed by using an automated qualitative enzyme-linked fluorescent immunoassay (bioMérieux, Marcy l’Etoile, France) and a quantitative indirect immunofluorescence test (Bion, Des Plaines, IL). In three patients, measles virus RNA was isolated from a punch of white matter obtained in the course of Ommaya reservoir implantation at SSPE onset. RNA was purified by the guanidinium-thiocyanate-phenol-chloroform method, and genetic characterization of measles virus was performed by sequencing the 450 nt from the carboxyl terminus of nucleoprotein (N) gene and a 377-bp fragment of the H gene (*9*). Viral fragments were reverse-transcribed with omniscript and sensiscript enzymes, and amplified with Hot Start Taq DNA Polymerase (QIAGEN GmbH, Hilden, Germany). Purified polymerase chain reaction products were labeled with DyET terminators and analyzed in an automatic capillary DNA sequencer (Amersham Biosciences, Piscataway, NJ). Comparisons were made with reference measles virus strains (*4*). Amino acid sequences were inferred. Nonsynonymous versus synonymous mutations (ω) and transitions versus transversions (κ) ratios were calculated. Sequences were aligned with ClustalX software, while phylogenetic analysis was performed by distance methods with Phylip software package v. 3.5c (*10*,*11*). Sequences derived from this study were submitted to GenBank.

## Discussion

Clinical, epidemiologic, and laboratory findings of eight SSPE patients are described in the Table. Mean age at SSPE onset was 54 months (range 40–75 months), and the mean lag period was 48 months (range 33–68 months). Most patients (six of eight) resided in the greater Buenos Aires metropolitan area, one from the northeastern region and the other from the northwestern region. All patients met at least clinical, EEG, and CSF antibody titer criteria defined by Dyken (*8*). In particular, patient 4 (at 6 months of age) had a clinical diagnosis of measles in September 1998 during the outbreak. Six months later, the patient received Measles Mumps Rubella (MMR) vaccine according to the official immunization schedules. For patient 5, clinical diagnosis of measles was made at 10 months of age in September 1998 during the outbreak. At that time, he also had varicella; 2 months later, the patient received the MMR vaccine. Patient 6 was born in November 1997 and had no previous history of measles infection. She also received antimeasles vaccine at 6 months of age during the measles outbreak when the vaccination age was lowered.

**Table:** Clinical, epidemiologic, and laboratory findings for eight pediatric subacute sclerosing panencephalitis (SSPE) patients^a^

Patient	SSPE1	SSPE2	SSPE3	**SSPE4**	**SSPE5**	**SSPE6**	SSPE7	SSPE8
Date of birth	Jan-98	Feb-98	Jul-98	Mar-98	Nov-97	Nov-97	Sep-96	Nov-97
Date of measles disease	Sep-98	Sep-98	May-99	Sep-98	Sep-98	N/A	Jul-97	Jul-98
Date of SSPE onset	May-01	Dec-01	Feb-02	Jun-02	Aug-02	Sep-02	Jan-03	Feb-03
Geographic location	GBA	GBA	NW	GBA	GBA	GBA	NE	GBA
CSF antibody titers	1:1600	1:1600	1:3200	1:3200	1:400	1:400	1:1600	1:400
Serum antibody titers	>1:3200	>1:3200	1:12800	>1:3200	>1:3200	1:12800	N/A	1:400
Initial diagnosis	Progressive myoclonic epilepsy	Acute disseminated encephalo-myelitis	Progressive myoclonic epilepsy	Neuronal ceroid lipofucsinosis	Lennox Gastaut syndrome	Progressive myoclonic atonic epilepsy	Chorioretinitis	Ataxia

^a^Molecular studies were done for SSPE patients highlighted in bold type. N/A, not available; GBA, greater Buenos Aires metropolitan area; NW, northwestern region from Argentina; NE, northeastern region from Argentina; CSF, cerebrospinal fluid.

### Molecular Findings

**Figure 1:**
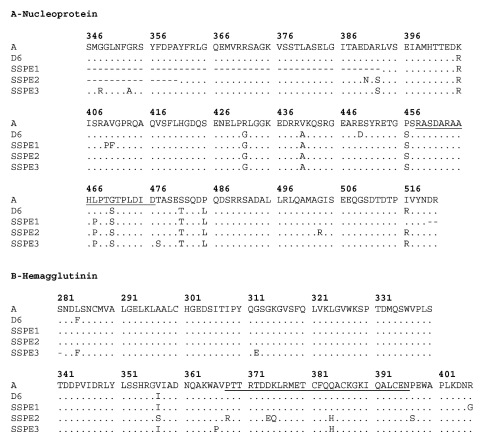
Replacement changes found in N and H genes from subacute sclerosing panencephalitis patients. Comparisons were made with D6 genotype reference strain (New Jersey, USA/94/1). Sample Buenos Aires.ARG/21.98 was taken as a consensus sequence for the 1998 Argentine outbreak (ARG21.98). Numbers indicate the position in A-nucleoprotein and B- hemagglutinin protein, respectively. Dots designate the same residue as genotype D6. Nonsilent changes are represented by single letter amino acid code. Antigenic sites are underlined.

The 450 nt from the carboxyl terminus of N gene and a 377-bp segment from H gene from three SSPE cases were analyzed and amino acid sequences were inferred. When compared to Edmonston strain, not only were a higher number of mutations found in N gene (36 vs. 19) but also more replacement changes (18 vs. 12) and a higher κ ratio (2.1 vs. 1.2) as compared to H gene. However, we compared the ω ratio and found it to be higher for H gene (1.7 vs. 1). Fixed replacements were detected at position 357 in H protein and at position 467 in N protein. Variability was detected in both genes. Changes found in SSPE samples related to D6 genotype, and the consensus sequence from last Argentine outbreak are summarized in Figure 1.

**Figure 2:**
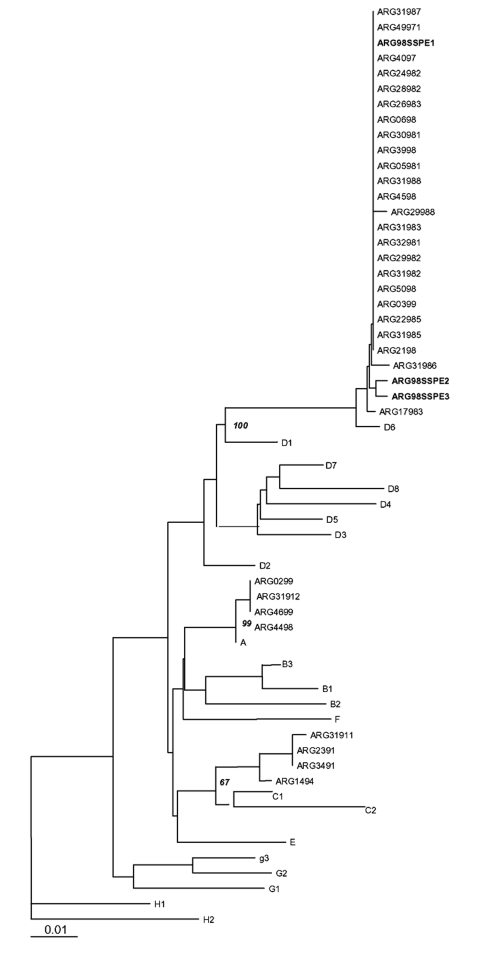
Phylogenetic relationships of subacute sclerosing panencephalitis (SSPE) strains. The neighbor-joining unrooted tree was plotted with Treeview 1.5.2. Reference measles virus strains are described (*2*). Wild-type (genotype C1 in 1991 and D6 in 1998) as well as post-vaccinal cases (genotype A) from the last two Argentine outbreaks were included (GenBank accession no. AF263841, 43, 44, 46, 52) (*7*). SSPE strains are highlighted in bold type (GenBank accession no. AY253332–37)

Distance methods were applied, and the matrix rendered similar results for both analyzed genes. The tree was built with measles virus genotype reference strains and Argentine strains previously characterized from 1991 and 1998 outbreaks. Phylogenetic relationships clustered the three SSPE strains with the wild-type D6 genotype. SSPE samples were strongly associated with wild-type D6 samples from the 1998 outbreak, supported by a bootstrap value of 100 out of 100 pseudoreplicates done. Divergence from genotype D6 (NJ-1 strain) was <2.2% in the N gene and <2.4% in the H gene. The unrooted tree for the carboxyl terminus of N gene was plotted (Figure 2).

## Conclusions

The measles vaccine was included in the regular immunization schedule in Argentina in 1978. Despite vaccination, several disease outbreaks have occurred (*12*). Although we had previously performed a thorough molecular description of acute measles outbreaks, genetic characterization of SSPE has not yet been reported in Argentina (*9*).

SSPE results in widespread destruction of brain tissue, including both gray and white matter. Infectious virus likely reaches the brain at the time of the original systemic spread of measles virus, where the virus becomes clonal, disseminating gradually throughout the nervous system from the point of entry (*13*). High levels of antimeasles antibodies are found both in serum and CSF, and their relative titers in the two compartments indicate intrathecal synthesis of immunoglobulins.

Measles virus in SSPE is characterized by a low expression of viral envelope proteins as a result of mutational events. Among such proteins, H is an attachment protein that mediates binding to cells and contributes to cell-to-cell fusion. The V357I mutation was one of the fixed changes that we had detected in the 1998 outbreak, but one of the SSPE cases (SSPE5) had a nonconservative V357S change at the same position (from hydrophobic to polar without charge) (*9*). This finding indicates that this particular position may be under strong positive selection supported by a high ω=1.7 and a low κ=1.2. Although this fragment is a relatively small portion of the H gene, it contains a major antigenic surface determinant (aa368–396), which may be relevant for neurovirulence, as well as three linear epitopes containing conserved cysteines 381, 386, and 396. SSPE5 presented 5 replacement changes; one shared with SSPE6 (P368R, D374E, K375Q, Q384H, and P397S) (*14*,*15*).

On the other hand, the N protein is more closely linked to viral protein interactions and thus has less constraints to vary as demonstrated by a higher κ (2.1) and lower ω (*1*) denoting neutral selection. Nevertheless, the B-cell epitope (aa457–476) showed the conservative L467P change in SSPE samples as well as in 1998 outbreak samples, differing from the D6 reference strain (NJ-1) (*16*).

Molecular data from the 1998 Argentine outbreak showed that the virus belonged to the D6 genotype and that analyzed regions were highly homogeneous and almost identical to other D6 strains isolated in South America (*9*). Accordingly, a single chain of transmission could be responsible for the spread of the genotype from European countries to Brazil and then to Argentina (*17*). The last Argentine outbreak began in July 1997 in the northeastern region of the country, bordering on Brazil; had its peak in greater Buenos Aires metropolitan area in July 1998; and ended in the northwestern region bordering on Bolivia in May 1999. For that reason, a displacement in time of acute measles and SSPE onset can be observed in SSPE3 and SSPE7.

Children in whom SSPE developed were born during the last measles outbreak and reached 6 months of age when the outbreak was at its peak. They acquired measles when they were 8 months of age (range 6–10 months), and SSPE was not detected among patients >1 year of age at the time of acute infection; however, underdiagnosis is a permanent challenge. Time lag between acute and SSPE onset was approximately 4 years, according to the reported data, but the rate for developing SSPE was higher than previously described (*8*).

Phylogenetic analysis of three SSPE cases from the last outbreak clustered with D6 genotype that circulated in Argentina in 1998. Although the original sequence of the wild-type virus that caused acute infection is unknown, we have a consensus sequence that summarizes the outbreak; therefore, we infer that changes may have occurred since then and contributed to the development of SSPE. Although all three patients had been immunized according to the schedule, vaccinal strains were not detected in brain tissue. These results agree with those recently reported for SSPE patients in the United Kingdom and Papua, New Guinea (*18*,*19*). Our data show that these three patients had been infected with wild-type circulating D6 virus before immunization. This primary measles virus infection in nonimmunized infants may be the leading cause of the high rate of SSPE inferred from our data. After brain tissues of deceased adults were randomly autopsied, Lawrence et al. raised the possibility that brain measles infection does not invariably lead to neurologic disease caused by measles virus. Therefore, neurologic disease mediated by measles virus may depend on the primary infection age (*20*). Although the basis for measles-associated neurologic disease is unclear and more thorough molecular studies need to be performed, our findings contribute to worldwide efforts in molecular characterization of SSPE strains and aim to increase awareness among physicians to improve diagnosis at early stages.
